# Effect of drying on the physical and chemical properties of faecal sludge for its reuse

**DOI:** 10.1016/j.jece.2019.103652

**Published:** 2020-02

**Authors:** S. Septien, S.W. Mirara, B.S.N. Makununika, A. Singh, J. Pocock, K. Velkushanova, C.A. Buckley

**Affiliations:** a Pollution Research Group, University of KwaZulu-Natal, Howard College, 4041, Durban, South Africa; b Chemical Engineering, University of KwaZulu-Natal, Howard College, 4041, Durban, South Africa

**Keywords:** Faecal sludge, Drying, Nutrient content, Calorific value, Thermal properties

## Abstract

This work explores the effect of drying on the chemical and physical properties of faecal sludge, and evaluates the reuse potential of the dried material. For the purpose of this study, the nutrient content, calorific value and thermal properties were determined for faecal sludge samples dried at different moisture contents and under different drying and operating conditions. The results show that drying does not affect the nutrient content and calorific value, but it induces modifications of the chemical form of nitrogen and the thermal properties. The dried product was demonstrated to be suitable for reuse as an agricultural product and biofuel. In agriculture, dried faecal sludge could be used as an organic fertilizer with a particularly high phosphorous content and a slow release of nitrogen and phosphorous. As a biofuel, dried faecal sludge would have similar characteristics as wood.

## Highlights

• Drying does not affect the nutrient content and calorific value of faecal sludge.
• Drying leads to a modification of the chemical form of nitrogen in faecal sludge.
• Moisture removal during drying decreases the thermal conductivity and heat capacity.
• Dried sludge is heated at a faster rate compared to when it is wet.
• Dried faecal sludge has the potential to be reused as organic fertilizer or biofuel.

## Graphical abstract

**Figure:**
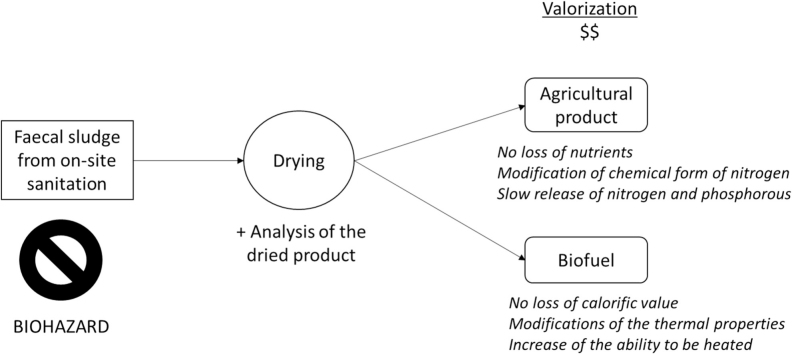

## 1 Introduction

According to figures from the World Health Organization [1], approximately 2.3 billion people in the world lack basic sanitation services, leading to contamination and health related issues. Worldwide efforts have been made in order to tackle the lack of sanitation that affects mostly poor regions. The improvement of sanitation was part of the agenda from the Millennium Development Goals [2], led by the United Nations in order to fight poverty and inequality. After the expiration of this initiative in 2015, the Sustainable Development Goals focused on provision of access to improved sanitation as one of the objectives to accomplish for 2030 (SDG 6). In support of the global efforts to overcome the global sanitation crisis, the Bill & Melinda Gates Foundation (BMGF) initiated the “Reinvent the Toilet Challenge” (RTTC) programme in 2011, which supports the development of innovative technologies that lead to affordable, sustainable and hygienic sanitation services, with the recovery of valuable resources from human excreta [3].

Faecal sludge being a hazardous material with high pathogen content, treatment of this type of waste is imperative for its disinfection and resource recovery. Different end-products can be obtained from faecal sludge, depending on the type of treatment process that it has been subjected to, as pointed out by Ronteltap et al. [4]. One of the possible reuse routes is in agriculture as fertilizer or soil-conditioner. Agriculture could be perceived as the most natural manner to valorise faecal sludge, as 60–70% of the nutrients from the crop fields are estimated to be found in human excreta [5]. Indeed, human excreta has a rich composition in nutrients that are not assimilated by the body during digestion. According to Wolgast [6], a person produces excreta sufficient enough to grow 250 kg of cereal per year, which covers their annual feeding requirements. The use of excreta in agriculture can decrease the need for synthetic fertilizers, leading to more sustainable agricultural production [7]. Apart from providing nutrients, excreta can improve the structure and water-holding capacity of the soil, reduce pests and neutralize soil toxins and heavy metals, which cannot be achieved through the use of synthetic fertilizers [8,9]. This can be particularly useful in the context of tropical soils, which require extensive conditioning [10]. However, the use of human excreta in agriculture still has social stigma and acceptance barriers to overcome, as noted by Cofie et al. [11].

Another possibility to reuse faecal sludge is as a biofuel. High heating values of 17 MJ/kg have been reported for faecal sludge in Kumasi (Ghana), Dakar (Senegal) and Kampala (Uganda) by Muspratt et al. [12]. These values are similar to those exhibited for biomasses such as coffee husks (16 MJ/kg), firewood (16 MJ/kg) and sawdust (20 MJ/kg). The Omniprocessor plant, designed by Janicki Bioenergy under the RTTC, can treat 12.3 m^3^ of sludge per day by incinerating it and produce 150 kW of electricity [13]. Some of the reinvented toilets from the RTTC are based on the combustion or smouldering of the solid fraction of the excreta: the “Nano-Membrane” toilet from Cranfield University [14,15]; the “Sanitation NoW” unit from Toronto University [16]; “A Better Toilet” from Research Triangle Institution [17]; “Firelight Toilet” from Janicki Bioenergy [18]. In addition, faecal sludge could be co-incinerated with coal in power plants or for industrial applications as cement kilns, similarly to sewage sludge [19,20]. As an alternative to combustion, faecal sludge can be turned into biochar by pyrolysis [21,22] or hydrothermal carbonization [23,24]. Biochar is a high value product that can be employed in several applications, such as fuel for heat or power generation, cooking fuel, soil-conditioner in agriculture and adsorbent in the pollution treatment industry.

Further products can be obtained from the treatment of faecal sludge, as a building material. Dried faecal sludge could be incorporated in the manufacturing of cement and bricks, and in the production of clay-based materials. Jordan et al. and Lin et al. have shown that faecal and sewage sludge have similar qualities to traditional construction materials such as limestone and clay [25,26]. Faecal sludge can also be used to feed animals, such as black soldier fly that can be sold subsequently as protein source in the market [27].

In all the aforementioned reuse options, drying is an important step in the treatment of faecal sludge. Faecal matter is a moist solid with a high pathogen content. In the eThekwini municipality (Durban, South Africa), the moisture content of faecal sludge from pit latrines and urine diversion dry toilets averages between 80 % wet basis (4 kg of moisture per kg dry solid) and 60 % wet basis (1.5 kg of moisture per kg of dry solid), respectively [28]. Several types of pathogens can be found in faecal sludge, such as bacteria, viruses, protozoa and helminths [29]. Drying provokes the removal of moisture by the application of heat, which reduces the mass and volume of the waste, and thus lowers the costs associated with transport and storage. In addition, the combined effect of heat and moisture removal during drying destroys the pathogenic organisms. For the thermochemical treatment of sludge (namely combustion, smouldering, pyrolysis), the removal of moisture until a certain content is needed in order to avoid the failure of the process and, if possible, to achieve a net positive energy balance for energy recovery.

The typical practice for faecal sludge dehydration consists of drying beds, where a thick layer of sludge is spread into a surface and exposed to solar irradiance for evaporation. The leachate percolates through the bed and passes through different filtration media, from where it is further treated or discharged into a water body. Nonetheless, this type of technology can take several days for moisture removal and the removal of pathogens is not guaranteed [11]. In order to be able to handle a higher throughput of faecal sludge, some treatment plants have opted to install contact or convective thermal driers in the process chain, such as the plant from Pivot in Kigali, Rwanda [30,31] and that from Tide Technocrats in Bangalore, India [32]. In some of the reinvented toilets previously cited, a drying system was integrated in order to dry the sludge before combustion. The eThekwini municipality (Durban, South Africa), with its industrial partner Particle System Separation, has developed an infrared dryer, ‘LaDePa’ (from ‘Latrine Dehydration Pasteurization’), for the disinfection and drying of faecal sludge from approximately 30,000 ventilated improved pit (VIP) latrines [33]. This process produces pasteurized and dried pellets that are planned to be sold as agricultural product. Emerging drying technologies, such as microwave driers, are also under development and testing [34].

Currently there is poor knowledge about faecal sludge drying in literature. The changes of the chemical and physical characteristics of faecal sludge during drying have not been yet explored, whereas this has important repercussions on the reuse of the dried product. In the case of similar materials as sewage sludge and manure for which the number of publications is considerably more important, only two studies were found on this respect. One deals with the effect of drying on the phosphorous content of manure [35] while the other investigated the effect of drying temperature on the heating value of sewage sludge [36].

In order to fill in this lack of knowledge, this study aims at characterizing the evolution of faecal sludge chemical and physical properties during drying. The feedstock was faecal sludge collected from VIP latrines, in the eThekwini municipality. Two methods of drying were employed, namely convective and infrared drying. The nutrient content, calorific value and thermal properties were measured at different stages of drying.

## 2 Material and methods

### 2.1 Faecal sludge sample collection and preparation

The samples used in the present work were faecal sludge collected during pit emptying of VIP latrines in the peri-urban areas of the eThekwini municipality. The samples were obtained from a few pit latrines. For each case, the samples were collected randomly from different depth and cross section positions of a pit during its emptying, following the procedure from Zuma et al. [37], and the composites were mixed to give an average representation of the pit content. It should be noted that the faecal sludge is highly heterogeneous and its characteristics can vary significantly depending on various factors such as the local population habits and socioeconomic situation, position of the pit with respect to the water table, type of construction of the pit basement… In our case, the samples presented the average moisture content of VIP sludge within the eThekwini municipality, which is around 80 % wet basis according to Zuma et al. [37], and had the typical appearance of the sludge found in the VIP latrines from the municipality.

Once transported to the laboratory, the sludge was sieved using a 5 mm grid in order to remove detritus such as plastics and textiles. Thereafter, the sieved sludge was stored in a cold room at 4 °C, in order to preserve the samples for experiments by minimizing any biological degradation and/ or physiochemical modification.

### 2.2 Description of the drying apparatus

#### 2.2.1 Convective drying rig

**Fig. 1:**
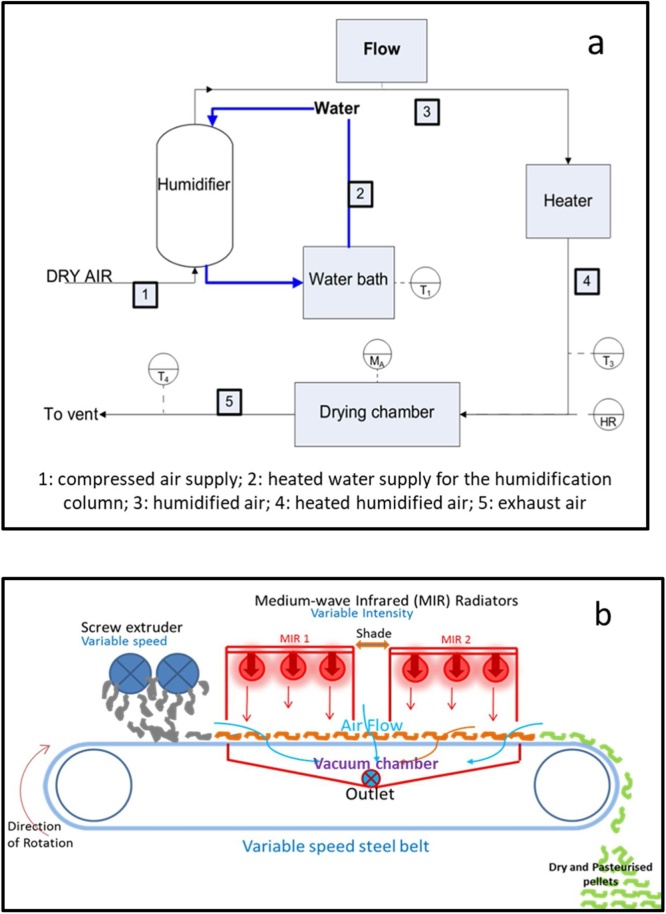
Schematic diagram of the convective drying rig (a) and the infrared drying rig (b).

In the convective drying rig, faecal sludge was dried by the means of a heated airflow. Fig. 1a shows the scheme of the convective drying rig. The apparatus is composed of three distinct sections: humidification, heating and drying section.

Compressed air was introduced in the drying rig, and the flowrate was controlled using a globe valve. In the first section, the air stream was humidified in a packed column where it was in contact with a counter-current liquid water flow set at a given temperature. In the second section, the humidified air was heated to the drying temperature by flowing over electric heating coils inside a chamber. In the third section, the hot humidified air was introduced into the drying chamber where the sludge was dried. In this section, the sample was situated on a hanging support linked to a precision weighing strain gauge load cell for the measurement of the mass. The measurements were continuously transmitted to a computer and recorded at a user specified time interval, in order to track the sample mass with time. Air temperature, flow rate, humidity and sample mass were constantly monitored and the values logged in a computer.

#### 2.2.2 Infrared dryer

The infrared dryer is a bench scale replicate of the full-scale LaDePa machine [33], with a size reduction of approximately 10:1. A schematic diagram of the bench scale LaDePa is shown in Fig. 1b.

The operation of the bench scale LaDePa is very similar to the full-scale machine. Pellets were formed by extrusion and placed onto the porous steel conveyer belt transporting the pellets into the heating zone. There, the pellets were exposed to thermal radiation from two medium infrared (MIR) emitters. The pellets left the belt via a discharge chute. Two vacuum chutes under the belt created an air stream in order to remove the evaporated moisture.

### 2.3 Drying of faecal sludge

#### 2.3.1 Convective drying rig

Drying was performed at different temperatures: 40, 60 and 80 °C. The air stream was maintained at a constant flowrate of 40 cm^3^/s and was not humidified for these experiments. Therefore, the air humidity during drying could be considered close to zero.

The sample was placed in the drying zone as a flat thin layer of 7 mm thickness (∼ 45 g) or as pellets of 8 mm diameters that were produced by a hand-held extruder (∼ 20 g). Note that the form of the sample did not lead to any considerable difference with respect to the chemical and physical properties. The sample was dried until when the sample mass stabilized, i.e. after reaching the equilibrium moisture content from which drying cannot further progress (equilibrium moisture content >10 % wet basis in this work).

#### 2.3.2 Infrared dryer

Experiments in the infrared dryer were carried out by varying the residence time and the infrared intensity. The residence time, controlled through the speed of the conveyor belt, was varied between 4 and 40 min. The infrared intensity was varied by adjusting the power supply of the emitters at 3.0, 4.7 and 6.0 kW, corresponding to temperatures in the drying zone of 85, 135 and 215 °C respectively (temperatures measured by a k-type thermocouple above the belt). The belt and the infrared emitters were separated by a distance of 115 mm. The suction area induced an air flowrate of 10.4 m^3^/min. The sample was placed on the conveyer belt as 8 mm pellets produced by a hand-held extruder.

### 2.4 Characterization of the sample

Different properties were measured for the initial sludge and samples after drying in the convective drying rig and the infrared dryer, based on the standard operating procedures developed by the Pollution Research Group [38].

The fertilizer potential of the processed faecal sludge was evaluated through chemical analysis. The content of phosphorus (P), potassium (K), calcium (Ca) and magnesium (Mg) was determined after digestion of the samples and analysis in a Microwave Plasma-Atomic Emission Spectrometer (MP-AES) model *Agilent 4100*. The digestion was performed by adding nitric acid to the sample and then placing the solution in a microwave digester model *Ethos 1 – Milestone*, heated to 130 °C for 1 h. The content of carbon (C), nitrogen (N) and sulphur (S) was measured in a CN analyser model *LECO TrueMac*. The content of molecular compounds, such as ammonium (NH_4_^+^), nitrates (NO_3_^−^), nitrites (NO_2_^−^) and phosphates (PO_4_^-3^), was analysed using the spectroquant *Nova 60-Merck*. The sample was prepared by blending it with water, centrifugation, recovering the liquid fraction and adding the commercial test kit specific for the measurement of the particular compound.

In order to evaluate the potential use of faecal sludge as a biofuel, the calorific value was measured in an oxygen bomb calorimeter model *Parr 6200*. This device is able to measure the heat of combustion after introduction of pure oxygen. The thermal conductivity, heat capacity and thermal diffusivity of the samples were measured using a *C-Therm TCi* thermal analyser. In this device, the sample was placed on a sensor which was heated over a short time. Through the thermal response of the system to the heat input, the thermal properties were determined.

### 2.5 Statistical analysis

All the tests were performed at least in duplicates, in order to verify the repeatability of the results. The measurement uncertainty was determined from the standard deviation of the repetitions using a Student’s t-distribution in a 90 % confidence interval.

## 3 Results and discussion

The chemical and physical characteristics of the sludge and pellets dried at different temperature in the convective drying rig are displayed in Table 1. Those obtained in the infrared dryer at different emitter intensities and residence times are shown in Table 2. The measurements that could not be done in replicates or presented a too high uncertainty bar, were not included in Table 1, Table 2. The uncertainty of the thermal diffusivity was not indicated, as its value was significantly low.

**Table 1:** Physical and chemical properties of the samples from the convective drying rig.

Sludge	Moisture content (% wet basis)	Nutrient content (g/g dry solid)										Calorific value (MJ/kg)	Thermal conductivity (W/m/K)	Heat capacity (kJ/kg/k)	Thermal diffusivity (m^2^/s) x 10^−8^
		C	N	P	K	Mg	Ca	NH_4_^+^	NO_2_^−^	NO_3_^−^	PO_4_^3−^				
Raw	80 ± 2	–	–	72 ± 8	8.9 ± 0.7	11 ± 1	37 ± 2	24 ± 4	–	1.6 ± 0.2	2.4 ± 0.7	–	0.568 ± 0.005	3756 ± 23	14.6
Dried at 40ºC	10 ± 2	337 ± 23	26 ± 4	71 ± 6	8.3 ± 1.1	11 ± 1	37 ± 2	4 ± 2	–	0.5 ± 0.1	2.3 ± 0.9	13.7 ± 0.2	0.044 ± 0.005	213 ± 13	28.0
Dried at 60ºC	5 ± 2	301 ± 77	25 ± 17	77 ± 5	8.9 ± 1.1	12 ± 2	44 ± 3	–	–	0.4 ± 0.1	2.5 ± 0.6	13.5 ± 0.4	0.045 ± 0.006	221 ± 8	27.5
Dried at 80ºC	5 ± 2	342 ± 102	25 ± 7	81 ± 5	8.6 ± 0.7	12 ± 1	50 ± 5	–	–	0.5 ± 0.1	2.1 ± 0.7	13.3 ± 0.5	0.043 ± 0.007	215 ± 13	27.0

**Table 2:** Physical and chemical properties of the samples from the infrared drying rig.

Sludge	Moisture content (% wet basis)	Nutrient content (g/kg dry solid)											Calorific value (MJ/kg)	Thermal conductivity (W/m/K)	Heat capacity (kJ/kg/k)	Thermal diffusivity (m^2^/s) x 10^−8^
		C	N	S	P	K	Mg	Ca	NH_4_^+^	NO_2_^−^	NO_3_^−^	PO_4_^3−^				
Raw	77 ± 0	–	–	–	107 ± 12	8 ± 1	12 ± 1	26 ± 8	–	13 ± 0	–	13 ± 0	17 ± 0.2	0.514 ± 0.002	4610 ± 8	12.7
Dried at 30 % MIR, 4 min	74 ± 1	335 ± 25	36 ± 3	9 ± 2	94 ± 35	6 ± 1	10 ± 5	24 ± 4	–	15 ± 0	–	–	–	0.372 ± 0.033	3930 ± 196	10.9
Dried at 30 % MIR, 9 min	71 ± 1	365 ± 33	40 ± 4	13 ± 6	104 ± 28	8 ± 1	12 ± 2	30 ± 14	–	16 ± 0	–	–	–	0.412 ± 0.012	4165 ± 65	11.3
Dried at 30 % MIR, 13 min	66 ± 1	319 ± 39	36 ± 2	10 ± 1	83 ± 25	7 ± 1	12 ± 1	23 ± 16	–	10 ± 0	–	13 ± 0	18.4 ± 1.0	0.270 ± 0.018	3179 ± 164	9.8
Dried at 30 % MIR, 17 min	61 ± 2	339 ± 19	35 ± 2	10 ± 1	99 ± 28	8 ± 2	12 ± 5	–	–	7 ± 0	–	–	21.6 ± 10.8	0.145 ± 0.007	1770 ± 102	9.4
Dried at 30 % MIR, 25 min	47 ± 2	320 ± 39	31 ± 3	7 ± 1	80 ± 9	7 ± 1	11 ± 1	26 ± 11	9 ± 9	7 ± 0	0.4 ± 0.0	11 ± 0	18.6 ± 0.8	0.062 ± 0.002	473 ± 28	15.2
Dried at 30 % MIR, 40 min	23 ± 6	375 ± 84	27 ± 8	–	76 ± 22	8 ± 3	13 ± 6	20 ± 6	–	3 ± 1	–	10 ± 3	16.1 ± 4.9	0.056 ± 0.001	366 ± 5	17.6
Dried at 50% MIR, 4 min	70 ± 0	338 ± 31	34 ± 4	11 ± 2	96 ± 38	10 ± 3	13 ± 7	24 ± 14	27 ± 10	14 ± 1	–	–	–	0.264 ± 0.022	3123 ± 209	9.7
Dried at 50 % MIR, 9 min	61 ± 1	340 ± 20	35 ± 1	11 ± 1	79 ± 36	8 ± 3	12 ± 9	–	–	17 ± 0	0.8 ± 0.5	–	19.8 ± 10.8	0.193 ± 0.007	2384 ± 82	9.3
Dried at 50 % MIR, 13 min	49 ± 2	304 ± 3	30 ± 3	7 ± 1	83 ± 31	9 ± 3	14 ± 9	–	9 ± 6	7.6 ± 0.3	–	11 ± 1	17.9 ± 1.0	0.123 ± 0.001	1247 ± 19	12.3
Dried at 50 % MIR, 17 min	33 ± 2	379 ± 16	30 ± 1	9 ± 1	88 ± 13	8 ± 1	14 ± 3	30 ± 17	–	4.0 ± 0.3	–	–	21.8 ± 8.8	0.059 ± 0.001	421 ± 12	16.2
Dried at 50 % MIR, 25 min	11 ± 1	380 ± 16	28 ± 1	10 ± 1	72 ± 26	8 ± 2	13 ± 3	25 ± 15	4 ± 1	1.2 ± 0.1	–	12 ± 2	15.7 ± 3.0	0.058 ± 0.001	404 ± 4	16.6
Dried at 80 % MIR, 4 min	61 ± 1	294 ± 85	30 ± 6	77 ± 25	77 ± 25	9 ± 3	13 ± 7	27 ± 7	15 ± 3	8.6 ± 0.1	–	–	18.9 ± 1.9	0.163 ± 0.001	2013 ± 19	9.9
Dried at 80 % MIR, 9 min	36 ± 1	339 ± 23	31 ± 2	84 ± 18	84 ± 18	10 ± 1	15 ± 10	30 ± 14	6 ± 1	2.3 ± 0.1	0.5 ± 0.0	11 ± 0	18.7 ± 0.5	0.068 ± 0.001	568 ± 19	13.8

The drying rates from the samples from this investigation can be seen in the work from Makununika in the case of the convective drying rig [39], and from Mirara and Septien et al. for the infrared drier [40,41].

### 3.1 Effect of drying on the nutrient content

#### 3.1.1 Elemental analysis

**Fig. 2:**
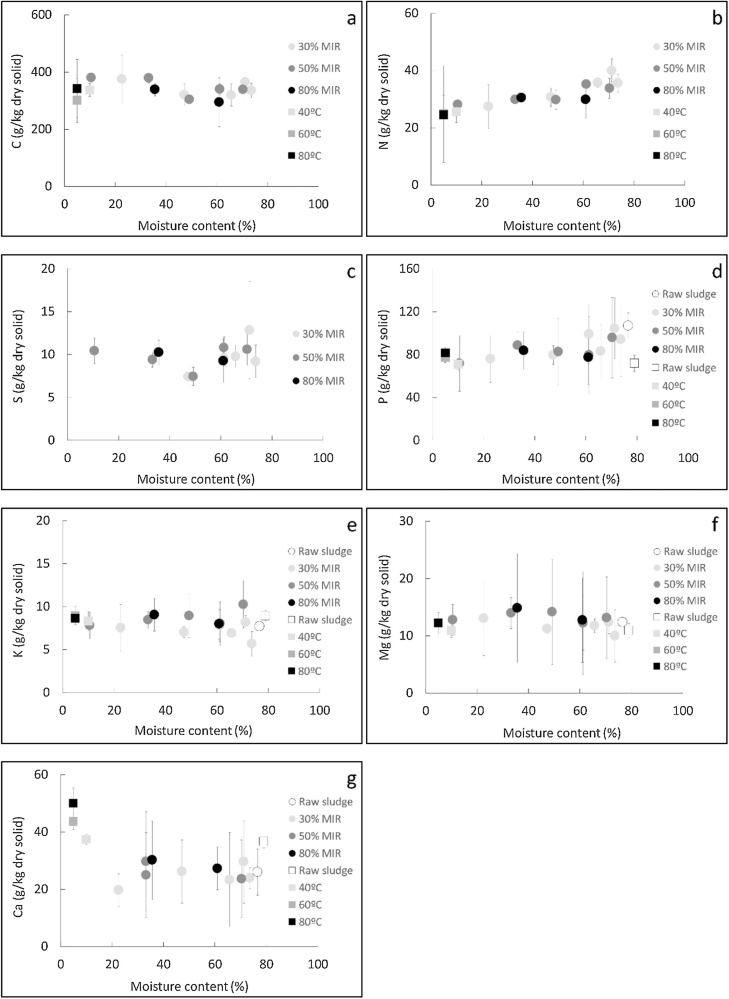
Elemental nutrient content versus moisture content for different operating conditions in the convective and infared drying rig: carbon (a); nitrogen (b); sulphur (c); phosphorous (d); potassium (e); magnesium (f); calcium (g).

The elemental nutrient composition of the sludge was plotted at different moisture content in Fig. 2. The graph combines the results from the convective and infrared drier.

It can be seen that, under the explored conditions, the nutrients concentration do not significantly change as a function of the moisture content, the operating conditions and the drying method. Therefore, drying did not affect the C, N, P, K, Mg and Ca concentrations in the pellets. The mean values for the C, N, S, P, K, Mg and Ca concentrations were 352, 31, 7, 85, 8, 12 and 30 g/kg of dry solid respectively.

Carbon was by far the major constituent, which could be expected as sludge is an organic material. Among the inorganic nutrients, the sludge had a particular high phosphorous content. It was higher than the typical concentration from manures and home compost, which varies from 0.5–25 g/kg of dry solid [[42], [43], [44]]. It is in the range of typical industrial fertilizers such as ammonium phosphate sulphate (85–170 g/kg), slag basic (50–80 g/kg), superphosphate single (70–90 g/kg) and urea ammonium phosphate (55–180 g/kg). The content of nitrogen and calcium of approximately 30 g/kg was higher than that from magnesium and potassium, which was roughly around 10 g/kg. The concentrations of these elements were in the range of the typical manure and compost nutrient content, which varies between: 5–50 g/kg of dry solid for N; 5–25 g/kg dry solid for K; 0.4–6 g/kg dry solid for S; 30–90 g/kg dry solid for Ca; 5–10 g/kg dry solid for Mg [[42], [43], [44], [45]].

#### 3.1.2 Molecular compounds composition

**Fig. 3:**
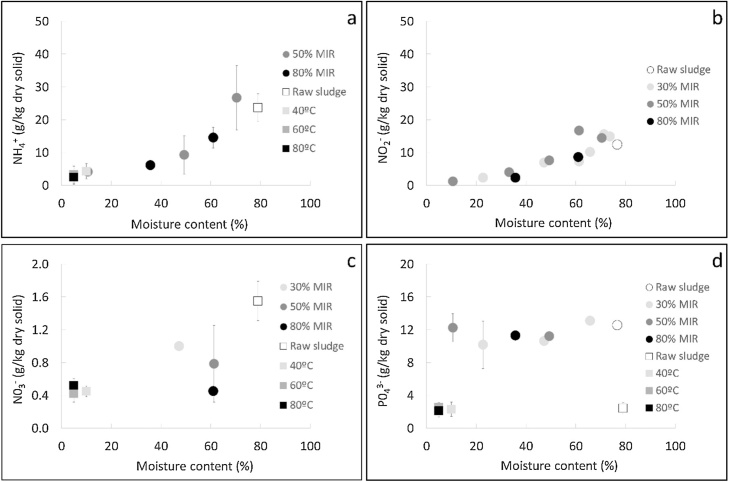
Molecular nutrient content versus moisture content for different operating conditions in the convective and infared drying rig: ammonium (a), nitrites (b), nitrates (c), phosphates (d).

It is important to determine the content of the nutrients in their molecular form, as plants assimilate some molecules more easily than others. For example, crops prefer the up-take of nitrogen as ammonia or nitrate depending on various conditions, such as the soil conditions and physiological characteristics of the plant [46]. Fig. 3 displays the content of ammonium, nitrates, nitrites and phosphates in the sludge, as a function of moisture content during drying in the convective and infrared apparatus. Note that these results may not account for the total concentration that could be found in the sludge, due to the limitations of the available extraction method. Indeed, in the experimental method conducted to determine the molecular compounds composition (see section 2.4), the sludge underwent an intense stirring and mechanical separation, in order to be able to extract as much as possible the compounds into the liquid fraction, which was thereafter analysed. Nevertheless, it was possible that a fraction of the compounds remained in the solid residue, particularly in the interior of the cells.

Among the nitrogenous compounds, ammonium was the major compound, followed by nitrites and finally nitrates, which were found in very small amounts. In the raw sludge, the sum of the ammonium, nitrates and nitrites concentration was around 42 g/kg dry solid, equivalent to a nitrogen content of 25 g/kg dry solid, which is close to the total nitrogen content (32 g/kg dry solid). This result suggests that the nitrogen in faecal sludge was mostly found as ammonium, nitrates and nitrites. Nonetheless, the concentration of these compounds decreased considerably as moisture was removed during drying. In the dried sludge, the sum of the ammonium, nitrates and nitrites concentration dropped to 4 g/kg dry solid, leading to a nitrogen content in these compounds considerably lower than the total content measured in the sludge (2 g/kg dry solid versus 32 g/kg dry solid). Considering that the total nitrogen content remained constant during drying, as noted in section 3.1.1, the decrease of the ammonium, nitrates and nitrites concentration could be result of changes of the chemical form of nitrogen during drying. In particular, it is suspected that the nitrogen created bonds with the dry bone structure, leading to the decrease of its chemical forms as individual molecules.

The phosphate concentration remained constant during drying, but the values differed between the sludge used for the experiments in the convective and infrared dryer (2 g/kg dry solid for the samples from the convective dryer and 10 g/kg dry solid for the samples from the infrared dryer). The content of the phosphorous present in the sludge as phosphate did not exceed 3 g/kg dry solid, which represented less than 4 % of the total phosphorous. Therefore, this result suggests that phosphorous was mainly found bounded to the dry bone structure, and in much lower proportions as phosphate. Drying seemed not to affect this partition in the sludge.

#### 3.1.3 Discussion

Dried sludge contains high amounts of carbon with a rich inorganic nutrient composition, which makes it suitable to use it in agriculture as organic fertilizer or soil conditioner. Under the explored conditions, the nutrient content of the dried sludge is in the range of typical organic fertilizers, such as manure and home compost, or even higher in the case of phosphorous. Dried faecal sludge is then an attractive alternative as a source of phosphorous, as for the synthesis of chemical fertilizers this element is usually extracted from non-renewable sources that are limited and then risk to be depleted.

In the present work, it was observed that drying does not affect the composition of the nutrient elements, but it can modify the chemical form of nitrogen in the sludge. In the raw material, nitrogen is found mainly as ammonium, nitrates and nitrites that can be drawn off relatively easily in the leachate from the sludge. In the dried material, nitrogen becomes difficult to remove as it is probably bounded to the solid structure. As an implication of this, it can be supposed that the dried sludge will slowly release the nitrogen if used for agricultural purposes. Slow-release fertilizers can minimize the potential nutrient losses by leaching or evaporation, allow to fertilize the soil for a longer period of time, lead to a more efficient nutrient uptake by the crops, require less number of applications and do not present a risk of burning for the roots of the plants [47]. This will contrast with the use of raw sludge in agriculture where the release of nitrogen could happen considerably faster, for example after irrigation or a rainfall, therefore nutrient leaching could be expected in this case.

Concerning phosphorous, only a small fraction is in the form of phosphates that can be removed in the leachate. Hence, most of the phosphorus must be strongly bounded into the solid matrix and can be assumed to be slowly released in the soil. No modification of the chemical form of phosphorus was detected during drying, on the contrary to nitrogen. This result is opposed to the findings with poultry manure, in which the distribution of water soluble and insoluble phosphorous was affected by drying [35].

### 3.2 Effect of drying on the calorific value

**Fig. 4:**
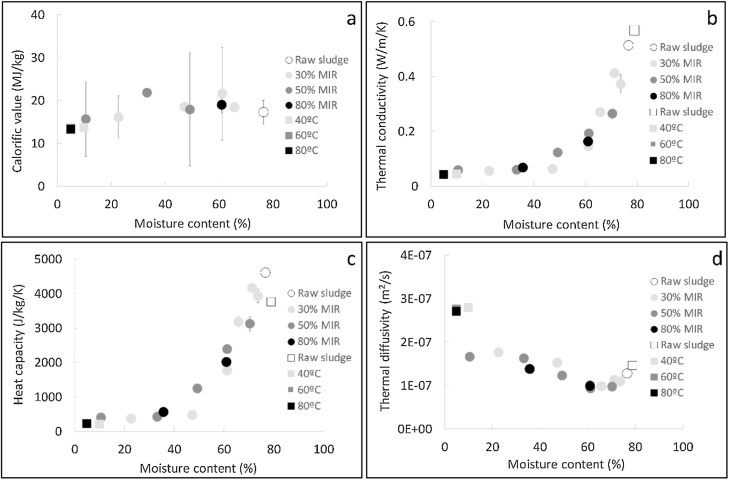
Calorific value (a) and thermal properties - thermal conductivity (b), heat capacity (c), thermal conductivity (d) - versus moisture content for different operating conditions in the convective and infared drying rig.

The calorific value on a dry basis (or high heating value) measured for the samples processed in the infrared and convective dryer is displayed as function of moisture content in Fig. 4a.

The calorific value was not significantly affected during either the infrared nor convective drying experiments at the different conditions. The average value, 18 MJ/kg dry solid, is similar to the calorific value of wood and some heating coals of low to medium rank, such as lignite, bituminous coal and peat (14–25 MJ/kg). It is approximately one third of the calorific value of common fossil fuels, such as diesel and natural gas (∼ 45 MJ/kg). Dried faecal sludge thus possesses a suitable calorific value to be used as a biofuel.

### 3.3 Effect of drying on thermal properties

Fig. 4b, c and d present the thermal properties of the samples dried in the infrared and convective drying apparatus, as a function of the moisture content.

#### 3.3.1 Effect on the thermal conductivity

Fig. 4b exhibits the variation of thermal conductivity during drying. It can be seen that the thermal conductivity decreased as moisture was removed, and it did not show any trend as a function of the drying conditions. Prior to drying, the faecal sludge thermal conductivity was approximately the same than that of pure water, 0.58 W/m/K, suggesting that its value was controlled by moisture. While drying up to a moisture content of 40 % wet basis, the thermal conductivity drastically decreased as a result of moisture removal. At moisture contents lower than 40 % wet basis, the thermal conductivity did not vary anymore and attained a constant value of 0.04 W/m/K. This value, approximately ten times lower than that of the fresh faecal sludge, is close to that of thermal insulating materials, such as wool (0.035 W/m/K), phenolic foam (0.020 W/m/K), and polystyrene (0.035 W/m/K), among others.

The low thermal conductivity exhibited by dried faecal sludge is an undesirable characteristic for a biofuel, as it leads to a more important resistance to heat penetration within the solid. The thermal conductivity of diesel, coal and wood (0.1 – 0.2 W/m/K) is higher than that of the dried faecal sludge, except for the thermal conductivity across the grain for some types of wood, e.g. balsa (∼ 0.055 W/m/K). The thermal conductivity of natural gas (∼ 0.03 W/m/K) is lower than that of the dried sludge, as expected for a gas.

On the opposite, a low thermal conductivity is a positive characteristic for the use of dried faecal sludge as a building material, as this implies a material with good thermal insulation characteristics.

#### 3.3.2 Effect on the heat capacity

The variation of heat capacity during drying is displayed in Fig. 4c. The trend was similar to that observed for thermal conductivity, i.e. a decrease of heat capacity as moisture was removed until reaching a constant value at moisture contents below 40% wet basis. This result could be also attributed to the diminution of the influence of moisture on the heat capacity as drying progressed. The heat capacity of the raw faecal sludge, 4600 J/kg/K, was close to the value of pure water, namely 4187 J/kg/K, whereas it was approximately 10 times lower in the case of dried sludge, i.e. 400 J/kg/K.

The decrease of the heat capacity after drying is positive for the thermal processes, as the temperature of the material can rise with a lower energy input. The value for the dried sludge is much lower than that of other common fuels, e.g. wood, coal, diesel and natural gas, varying between 1200 and 2900 J/kg/K.

#### 3.3.3 Effect on the thermal diffusivity

The thermal diffusivity refers to the ability of a body to conduct heat relative to its ability to store thermal energy. It is defined as the ratio of the thermal conductivity to the product of density and heat capacity. The thermal diffusivity for the samples at different moisture contents is shown in Fig. 4d.

The thermal diffusivities were in the order of 10^−7^ m^2^/s and exhibited a slight increase as the moisture content decreased. The thermal diffusivity of the dried solid was approximately 3 times higher than that of the raw sludge. As the heating rate of a material is directly related to the thermal diffusivity, it can be expected that dried faecal sludge would be heated faster than the wet sludge.

Compared to other fuels, the thermal diffusivity of dried faecal sludge (∼ 3·10^−7^ m^2^/s) is higher than fuels such as diesel, wood and coal (∼ 1–2·10^−7^ m^2^/s), but lower than natural gas (∼ 2.0·10^-5^ m^2^/s).

#### 3.3.4 Discussion

The dried faecal sludge has a great potential to be used as biofuel: a relatively high calorific value, which is similar to wood and some coal ranks; a thermal diffusivity in the same order of magnitude compared to common liquid and solid fuels.

As demonstrated by Hanson et al. in their study about the effect of thermal properties of high water content materials [48], the thermal conductivity and heat capacity of the raw faecal sludge were surely controlled by its high moisture content. During drying, the influence of moisture on the thermal properties diminished, leading to a decrease of the thermal conductivity and heat capacity, and was probably completly lost at a moisture content of 40 % from where the values of the thermal properties did not vary after further drying. As a result of these modifications, the thermal diffusivity was higher in the dried faecal sludge compared to the wet material, which implies a faster heating of the sludge when it is dried.

Under the explored conditions, the calorific value of faecal sludge was not affected by drying, even at the most severe conditions (infrared drying at 6.0 kW, leading to a temperature of approximately 215 °C, during 8 min). This differs from the results from the investigation of Vesilind and Ramsey where they found that the sewage sludge heating value decreased by drying temperatures higher than 150 °C [36].

If used as biofuel, it has to be considered that hazardous pollutants could be formed during the combustion of faecal sludge, as this material can present sulphur and nitrogen in its composition, as discussed in the previous sections. The implementation of operation units for gas treatment is then an option that should be taken into consideration in faecal sludge incineration plants, as a function of the composition of faecal sludge and the operating conditions of the plant.

## 4 Conclusions

Drying does not affect the nutrient content and the calorific value in faecal sludge, but it provokes some chemical and physical modifications, such as: change of the nitrogen chemical form; a decrease of thermal conductivity and heat capacity, leading to a higher thermal diffusivity. The dried pellets present an attractive nutrient composition for agricultural applications, particularly in terms of phosphorus and the probable slow release of nitrogen and phosphorous in the soil. The use of dried pellets as a biofuel is another interesting alternative because of the relative high calorific value and good thermal diffusivity of the material.

## Funding

This work was supported by the Bill & Melinda Gates Foundation [grant OPP1069575] and the South African Water Research Comission [grant K5/2137].

## CRediT authorship contribution statement

**S. Septien:** Conceptualization, Methodology, Validation, Formal analysis, Data curation, Writing - original draft, Visualization, Supervision, Project administration. **S.W. Mirara:** Conceptualization, Methodology, Validation, Formal analysis, Investigation, Visualization. **B.S.N. Makununika:** Conceptualization, Methodology, Software, Validation, Formal analysis, Investigation, Visualization. **A. Singh:** Methodology, Writing - review & editing, Supervision. **J. Pocock:** Methodology, Writing - review & editing, Supervision. **K. Velkushanova:** Conceptualization, Methodology, Writing - review & editing, Project administration. **C.A. Buckley:** Conceptualization, Methodology, Writing - review & editing, Supervision, Project administration, Funding acquisition.

## Declaration of Competing Interest

None.
